# Urinary proteome profiles associated with cognitive decline in community elderly residents—A pilot study

**DOI:** 10.3389/fneur.2023.1134976

**Published:** 2023-03-16

**Authors:** Yumi Watanabe, Yoshitoshi Hirao, Kensaku Kasuga, Kaori Kitamura, Kazutoshi Nakamura, Tadashi Yamamoto

**Affiliations:** ^1^Division of Preventive Medicine, Niigata University Graduate School of Medical and Dental Sciences, Niigata, Japan; ^2^Biofluid and Biomarker Center, Graduate School of Medical and Dental Sciences, Niigata University, Niigata, Japan; ^3^Department of Molecular Genetics, Brain Research Institute, Niigata University, Niigata, Japan

**Keywords:** urine, cognitive decline, dementia, biomarker, proteomics

## Abstract

Non-invasive and simple methods enabling easy identification of individuals at high risk of cognitive decline are needed as preventive measures against dementia. This pilot study aimed to explore protein biomarkers that can predict cognitive decline using urine, which can be collected non-invasively. Study subjects were selected from participants in a cohort study of middle-aged and older community-dwelling adults who underwent cognitive testing using the Mini-Mental State Examination and provided spot urine samples at two time points with an interval of approximately 5 years. Seven participants whose cognitive function declined 4 or more points from baseline (Group D) and 7 sex- and age-matched participants whose cognitive function remained within the normal range during the same period (Group M) were selected. Urinary proteomics using mass spectrometry was performed and discriminant models were created using orthogonal partial least squares-discriminant analysis (OPLS-DA). OPLS-DA yielded two models that significantly discriminated between the two groups at baseline and follow-up. Both models had ORM1, ORM2, and SERPINA3 in common. A further OPLS-DA model using baseline ORM1, ORM2, and SERPINA3 data showed similar predictive performance for data at follow-up as it did for baseline data (sensitivity: 0.85, specificity: 0.85), with the receiver operating characteristic curve analysis yielding an area under the curve of 0.878. This prospective study demonstrated the potential for using urine to identify biomarkers of cognitive decline.

## 1. Introduction

With the aging of the population, the number of people with dementia has become an important societal issue. Currently, more than 55 million people live with dementia worldwide, and there are nearly 10 million new cases every year (1). A recent study estimated an increase in the number of people with dementia to 152 million by 2050 (2).

Dementia is a disorder in which a person's previous level of cognition is severely declined and interferes with occupational, family, and social functioning (3). It is considered an acquired syndrome with multiple possible causes, such Alzheimer's disease (AD), brain cancer, and brain injury, and usually progresses slowly and chronically (4).

Studies have shown that intervention targeting a variety of modifiable risk factors may prevent or delay the onset of dementia (5–7). The identification of biomarkers that predict cognitive decline through non-invasive and easily testable screening methods is expected to enable effective approaches to identify high-risk populations for dementia.

Urine is one of the preferable biological fluids as a source of disease biomarkers because it can be collected easily and non-invasively. A biomarker is defined as a laboratory measurement that reflects the activity of a disease process (8). Approximately 30% of the proteins in urine are normally derived from plasma, and 70% are produced in the kidneys (9). Substances removed from the blood are excreted in the urine to maintain homeostasis in the body. Thus, urine can be a source of biomarkers that reflect changes in the body more sensitively than other biofluids. This is especially important in the early stages of disease, when the homeostatic mechanisms that maintain a stable environment in the body are still in effect and removing these harmful substances from the body in a variety of ways (10).

Urine is also a suitable source for proteomics analysis using mass spectrometry, which is currently the most common method for the discovery and identification of candidate biomarkers (11). The protein concentration in normal urine is very low. According to a recent study (12), in pooled samples from healthy individuals, the plasma protein concentration was 80 mg/ml while the urine protein concentration was 0.03 mg/ml. Nevertheless, thousands of proteins were identified in urine. The proportion of some major proteins, most notably albumin, is lower in urine than in plasma (13, 14), which also favors the identification of low-abundance proteins.

A recent study comparing the proteomes of plasma, cerebrospinal fluid (CSF), urine, and saliva revealed that the urine proteome shares many proteins with the other four body fluids (12). Interestingly, while the plasma proteome is enriched in complement-related proteins and integrin signaling-related proteins, the urine proteome was shown to be enriched in a wider variety of proteins, including axon guidance-related proteins, glycolytic system-related proteins, and acute phase response-related proteins (12). Although brain and urine appear to be far apart, urine may be a more sensitive reflection of pathologies related to neurodegeneration and inflammation.

Currently, studies on urine-based protein biomarkers of dementia and cognitive decline are limited. AD-associated neuronal thread protein (AD7c-NTP) has been reported to be specifically found in the brains of AD patients (15). A recent study demonstrated that urinary AD7cNTP concentrations in AD, MCI, and healthy controls were found to shift in order from high to low (16). Yao et al. found that urine from AD patients had significantly decreased levels of osteopontin and increased levels of gelsolin and insulin-like growth factor-binding protein 7 compared to healthy elderly subjects (17). We previously reported on the urinary proteome profiles of individuals with AD compared with age- and sex-matched controls without cognitive impairment (18, 19). However, these previous studies were cross-sectional in design and thus cannot predict the onset of AD or dementia.

Over the past 4 years, we have followed community-dwelling elderly individuals, testing their cognitive function with the Mini-Mental State Examination (MMSE) (20) and collecting urine samples. The present study aimed to compare the urinary proteome profiles of those with cognitive decline based on changes in MMSE scores over the study period with those whose cognitive function remained normal.

## 2. Materials and methods

### 2.1. Subjects

This study was approved by the human research ethics committee of Niigata University (approval numbers: 1836, 2018-0057, 2019-04545). All subjects were informed through a verbal consent process (21).

Subjects were selected from a subcohort (Sekikawa cohort) of the Murakami cohort, a population–based cohort study that targeted individuals aged between 40 and 74 years living in areas of northern Niigata Prefecture (Murakami region) (21), who underwent the MMSE (MMSE-J: the Japanese version of MMSE) (22) and provided a urine specimen at the baseline survey in 2014 or 2015 and at the follow-up surveys in 2019 and 2021 (18). At the baseline survey, 415 subjects took the MMSE and provided urine samples. Among these, 189 took the MMSE and provided urine samples at the follow-up survey. The average follow-up was 4.7 ± 0.8 years. Demographic characteristics of subjects are shown in Supplementary Table 1. Tombaugh et al. reported that a 4-point decrease in MMSE score over 5 years is a significant change (*p* < 0.05) (23), and Stein et al. reported that a change of 2–3 points in MMSE score over a time period of ~4.5 years is significant and reliable at the 90% confidence level (24). Therefore, subjects whose MMSE score decreased by 4 or more points during the study period and whose MMSE score was < 24 (i.e., suspected dementia) at follow-up were considered to have cognitive decline. 16 subjects had a decrease in MMSE scores of 4 or more points, with baseline scores ranging from 26 to 30 points. Of these, 7 had a score of < 24, which is considered suspected dementia at follow-up survey, and nine had a score in the range of 24 to 27, which is considered suspected mild cognitive decline (22). Consequently, 7 subjects were included in the cognitive decline group (Group D). MMSE scores for the Group D at baseline ranged from 26 to 30 points. For the control group (Group M), subjects who maintained cognitive function (i.e., those with MMSE scores of 28 or higher at both baseline and follow-up) were matched for sex and age with the Group D. The flowchart of subject selection is shown in the Supplementary Figure 1. MMSE scores for subjects of Group D and M are shown in Supplementary Table 2.

### 2.2. Urine sample collection and laboratory tests

Spot urine samples were obtained from subjects. No restrictions on diet, drinking, or exercise were required prior to urine sampling. Urine specimens were refrigerated in a cooler box immediately after collection and brought back to the laboratory within 8 h. Samples were then centrifuged at 1,000 g for 15 mins and the supernatant was stored at −20°C until use. Urine samples that were positive for urinary protein, sugar and/or occult blood by test strips (Hema-Combistix-long, Siemens Healthcare Japan) were excluded.

### 2.3. Protein extraction from urine samples

Urine proteins were extracted according to a previously reported sample preparation method for urine (25). In brief, 1 ml of urine was mixed with an equal volume of methanol with one fourth chloroform and mixed well for 5 mins. The sample was then centrifuged at 19,000 g at 25°C for 15 mins and the supernatant was discarded. Subsequently, 1 ml of methanol was added to the sample, which was mixed gently for 5 mins and then centrifuged at 19,000 g at 25°C for 15 mins. Finally, the supernatant was removed, and the obtained proteins were dried by air.

After precipitation, proteins were dissolved in 50 mM Tris-HCl (pH 8.0) buffer containing 8 M urea. For reduction and alkylation, proteins were treated with dithiothreitol and iodoacetamide, respectively, for 1 h. Following this, 1 μg of trypsin (Agilent, USA) was added to the sample and the mixture was incubated at 37°C for 16 h with shaking. The digested sample was purified with a C18 spin column (GL Science, Japan) according to the manufacturer's instructions. The eluted sample was dried using a VEC-260 vacuum dryer (Iwaki, Japan). The sample was then re-suspended in 0.1% formic acid and the peptide concentration was measured using a Nano drop 1,000 machine (Thermo). Samples were stored at −80°C until use.

### 2.4. Mass spectrometry analysis

The Fusion Tribird mass spectrometer was connected to an EASY nLC1000 system (Thermo Fisher Scientific, Inc. Bremen, Germany). Each sample (500 ng) was injected into the LC system on a trap column (2 cm × 75 μm Acclaim Pepmap 100 column) and separation column (12.5 cm × 75 μm NTCC-360) at 300 nL/min, with a 115 mins multistep gradient. Peptides were separated with 5%B for 5 mins, 25%B for 100 mins, and 35%B for 115 min with mobile phase A: water with 0.1% formic acid; mobile phase B: acetonitrile with 0.1% formic acid. The mass spectrometer was set to the positive ion mode in scan ranges for MS and MS/MS of 350–1,500 and 200–2,000 m/z, respectively. MS spectra were acquired with high resolution (120,000) and a 3-sec cycle time.

### 2.5. Quantification analysis

The human Uniprot protein sequence database (v2015-08; Homo sapiens 20,203 sequences) was searched with Proteome Discoverer 2.2 (Thermo Fisher Scientific, Inc. Bremen, Germany) directly using. RAW files with the SEQUEST algorithm. The following settings were applied: trypsin with two missed cleavages, mass tolerance of ± 10 ppm, fragment ion mass tolerance of ± 0.6 Da, and cysteine carbamidomethylation as a fixed modification. False discovery rate (FDR) was set to 1% at the peptide level. Label-free quantification (LFQ) was selected for quantification and the analysis was performed using the default consensus workflow of Proteome Discoverer.

### 2.6. DATA processing and bivariate analysis

Abundance of the master protein for each sample obtained by Proteome Discoverer 2.2 was used as the quantitative value. Samples from Groups D and M at the baseline survey were denoted as D1 and M1, respectively, and samples from Groups D and M at the follow-up survey as D5 and M5, respectively. Mass spectrometry analysis identified 830 ± 222, 859 ± 135, 1,048 ± 100, and 1,074 ± 96 proteins with quantitative value in the D1, M1, D5, and M5 samples, respectively. The 1,159 proteins identified in 4 or more samples in the D1, M1, D5, or M5 sample were included in the statistical analysis. Missing values were filled with 1/5 of the minimum value for each protein prior to statistical analysis. Wilcoxon's rank-sum test was used to compare levels of compounds between Groups D and M. SAS software was used for statistical analyses (release 9.13, SAS Institute Inc., Cary, NC, USA). P < 0.05 was considered statistically significant.

### 2.7. Multivariate analysis

To identify proteins which can discriminate between Groups D and M, principal component analysis (PCA) and orthogonal partial least-squares discriminant analysis (OPLS-DA) was applied using SIMCA software (version 17.0, Umetrics AB, Umea, Sweden) (26). Before analyses, data that were not normally distributed were logarithmically transformed accordingly with the automatic transformation criteria of the software. Data were then mean-centered and scaled to unit variance. First, PCA was tested using baseline data and follow-up data respectively. The resulting plots are shown in Supplementary Figure 2. OPLS-DA was then performed. OPLS-DA provides supervised clustering of subjects into two groups. The reliability of OPLS-DA models was determined by analysis of variance testing of cross-validated predictive residuals (CV-ANOVA). The cross validation was performed 7 times. R^2^ is a parameter that measures the goodness of fit while Q^2^ is another parameter that measures the predictive ability of the model. In an ideal model, the R^2^ and Q^2^ should be similar, meaning that each of the samples contributes equally and uniformly to the observed group separation. In reality, Q^2^ is always lower than R^2^; however, if Q^2^ is substantially lower than R^2^, the robustness of the model is poor, implying overfitting (26). Following the suggestion of Wheelock et al. (26). we reported number of components, R^2^, Q^2^, and CV-ANOVA *p*-value as model statistics of the final models. Variable importance in the projection (VIP) provides the influence of every variable in the model. A higher VIP value represents a stronger contribution to discrimination among groups. Variables with VIP >1 made above-average contributions to the model. Predictive VIP (VIPpred) is the predictive component of VIP. Receiver operating characteristic (ROC) curve analysis was performed with SIMCA software.

## 3. Results

Baseline ages of subjects were 71.3 ± 5.5 and 71.9 ± 5.3 years in Groups D and M, respectively. There were 4 males and 3 females in both groups. MMSE scores for Groups D and M were 27.7 ± 1.4 and 29.0 ± 0.6 points, respectively, at baseline (p = 0.05, Welch's *t*-test), and 21.0 ± 1.5 and 29.3 ± 0.8 points, respectively, at follow-up (*p* < 0.001, Welch's *t*-test). Differences between MMSE scores (i.e., follow-up score minus baseline score) in Groups D and M were 6.7 ± 1.4 and −0.3 ± 1.1 points, respectively (*p* < 0.001, Welch's *t*-test).

**Figure 1:**
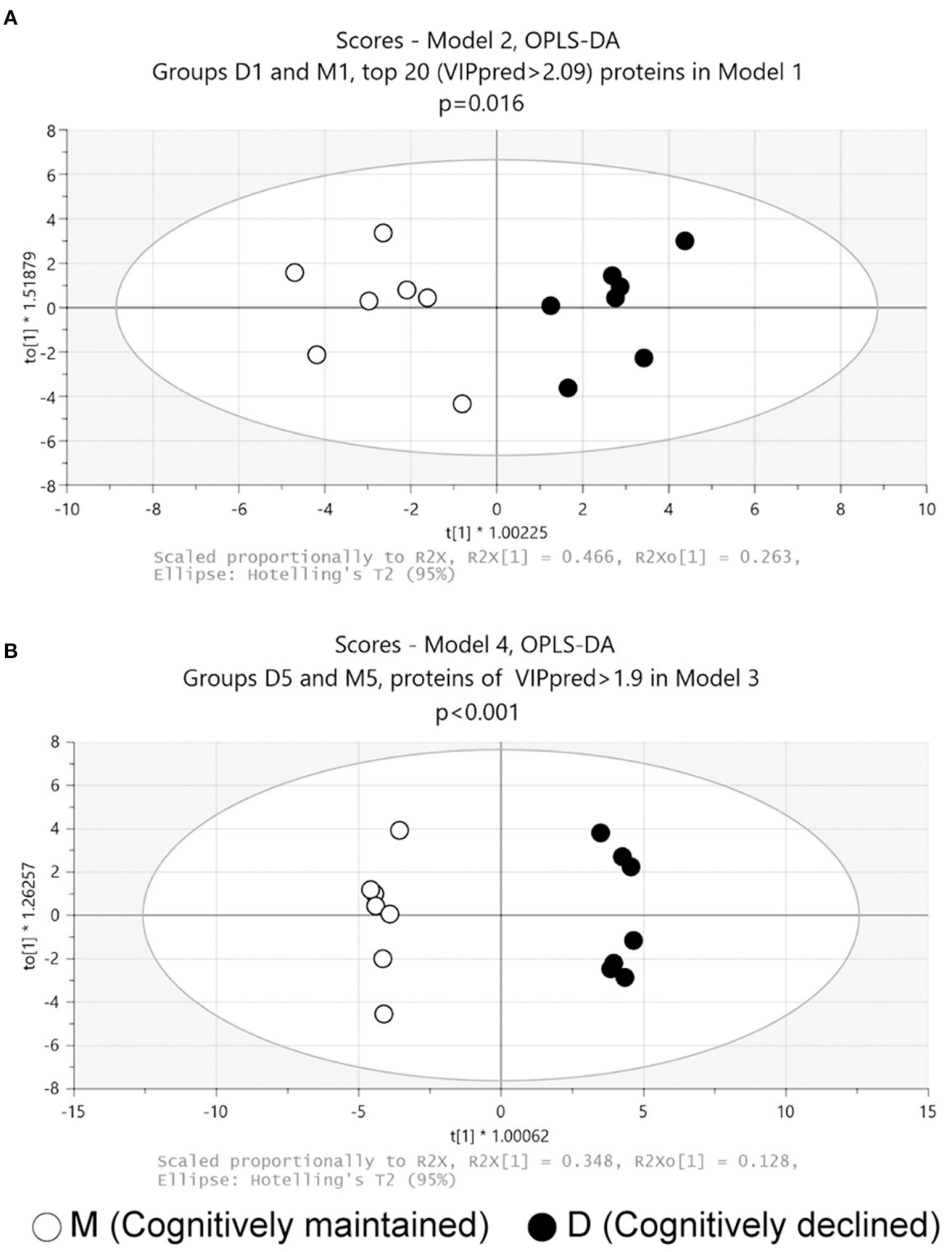
Score plots of OPLS-DA models. **(A)** Model 2: Model 2 was generated using the top 20 proteins based on the predictive VIP score of the OPLS-DA model using all proteins in the proteome data at baseline. **(B)** Model 4: Model 4 was generated with 54 proteins with predictive VIP >1.9 in the OPLS-DA model using all proteins in the proteome data at follow-up. *p* values obtained from CV-ANOVA are shown.

Discriminant models using OPLS-DA were first created from baseline data (i.e., D1 and M1 samples). The model using all proteins failed to discriminate significantly between D1 and M1 samples (Model 1: *p* = 0.44, CV-ANOVA), but a significant discriminant model was created with the top 20 proteins based on the predictive VIP score of Model 1 (Figure 1A, Model 2: 2 components, R^2^ = 0.85, Q^2^ = 0.71, *p* = 0.016 (CV-ANOVA); the list of proteins included in Model 2 are shown in Table 1).

**Table 1:** Proteins included in Model 2.

**Accession**	**Gene name**	**VIP pred in Model 1**	**Fold change in median**	***P*-value (Wilcoxon rank sum test)**
P13473	LAMP2	2.781	3.244	0.026
P36957	DLST	2.607	14.251	0.001
P22105	TNXB	2.518	3.309	0.026
P02763	ORM1	2.493	4.624	0.017
P19652	ORM2	2.469	9.870	0.011
P09668	CTSH	2.444	16.298	0.026
P40189	IL6ST	2.377	5.336	0.017
P01011	SERPINA3	2.372	2.579	0.097
Q99538	LGMN	2.334	5.000	0.070
P12273	PIP	2.283	8.060	0.072
P02751	FN1	2.280	1.823	0.053
P16112	ACAN	2.245	4.210	0.097
Q9UNZ2	NSFL1C	2.234	94.277	0.010
P54727	RAD23B	2.231	11.176	0.070
P52758	RIDA	2.197	32.259	0.005
O60888	CUTA	2.178	2.776	0.017
P05546	SERPIND1	2.173	9.924	0.002
Q9HC84	MUC5B	2.117	69.533	0.040
Q9BYE9	CDHR2	2.104	1.891	0.053
P04259	KRT6B	2.098	0.081	0.021

Top 20 proteins based on the predictive VIP (VIP pred) score in Model 1 using all proteins in the proteome data at baseline.

OPLS-DA was then performed on follow-up data (D5 and M5 samples). The analysis using all proteins failed to discriminate significantly between D5 and M5 samples (Model 3: *p* = 0.53, CV-ANOVA). A further analysis using 54 proteins with a predictive VIP score >1.9 in Model 3 significantly discriminated between D5 and M5 samples (Figure 1B, Model 4: 3 components, R^2^ = 0.99, Q^2^ = 0.91, *p* < 0.001(CV-ANOVA); the list of 54 proteins is shown in Supplementary Table 3).

**Figure 2:**
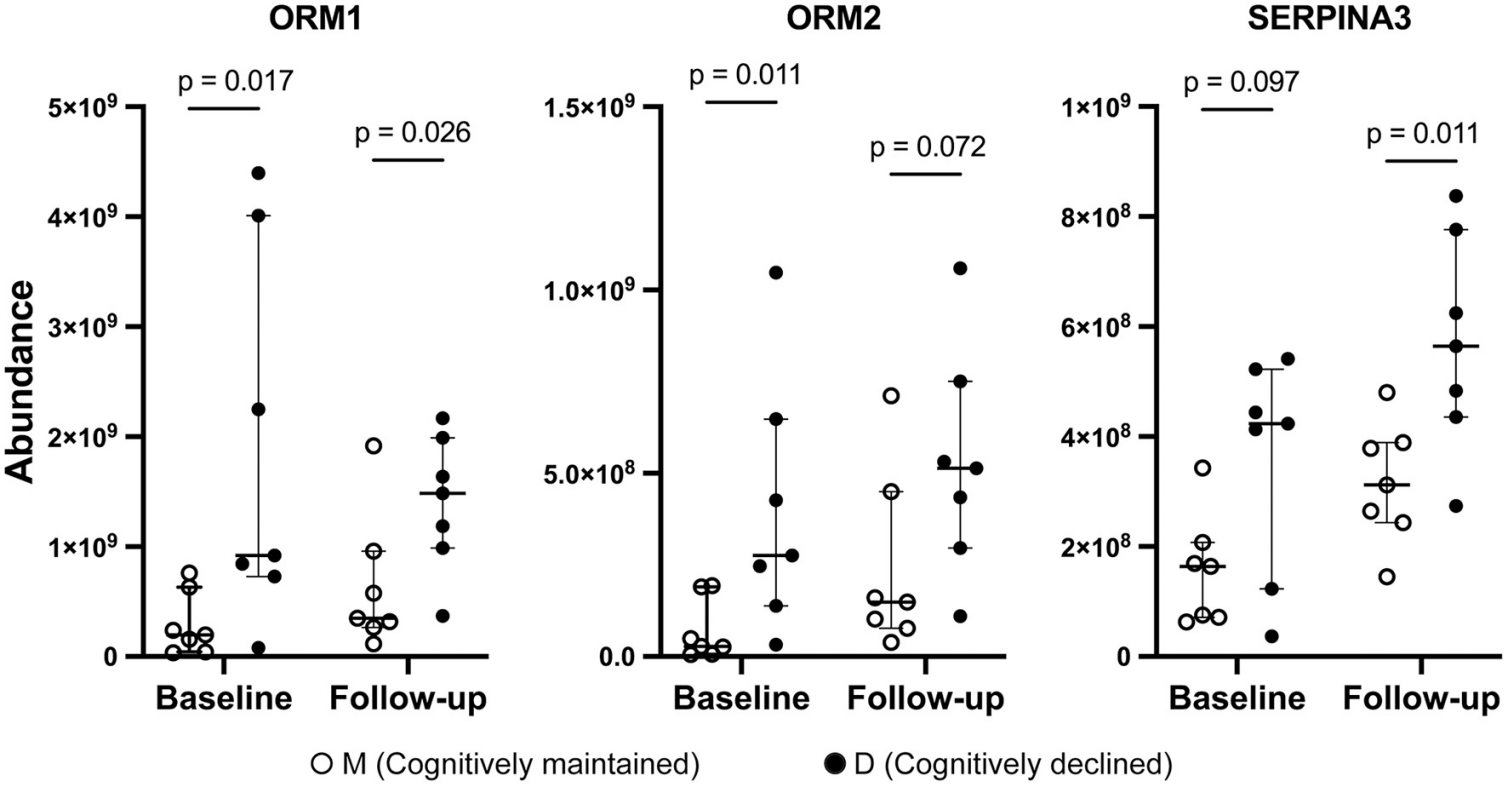
Abundance of ORM1, ORM2, and SERPINA3 by mass spectrometry. Solid circles: Group D, open circles: Group M. Bars and ranges represent median and interquartile range. *P*-values were obtained using the Wilcoxon's rank sum test.

**Figure 3:**
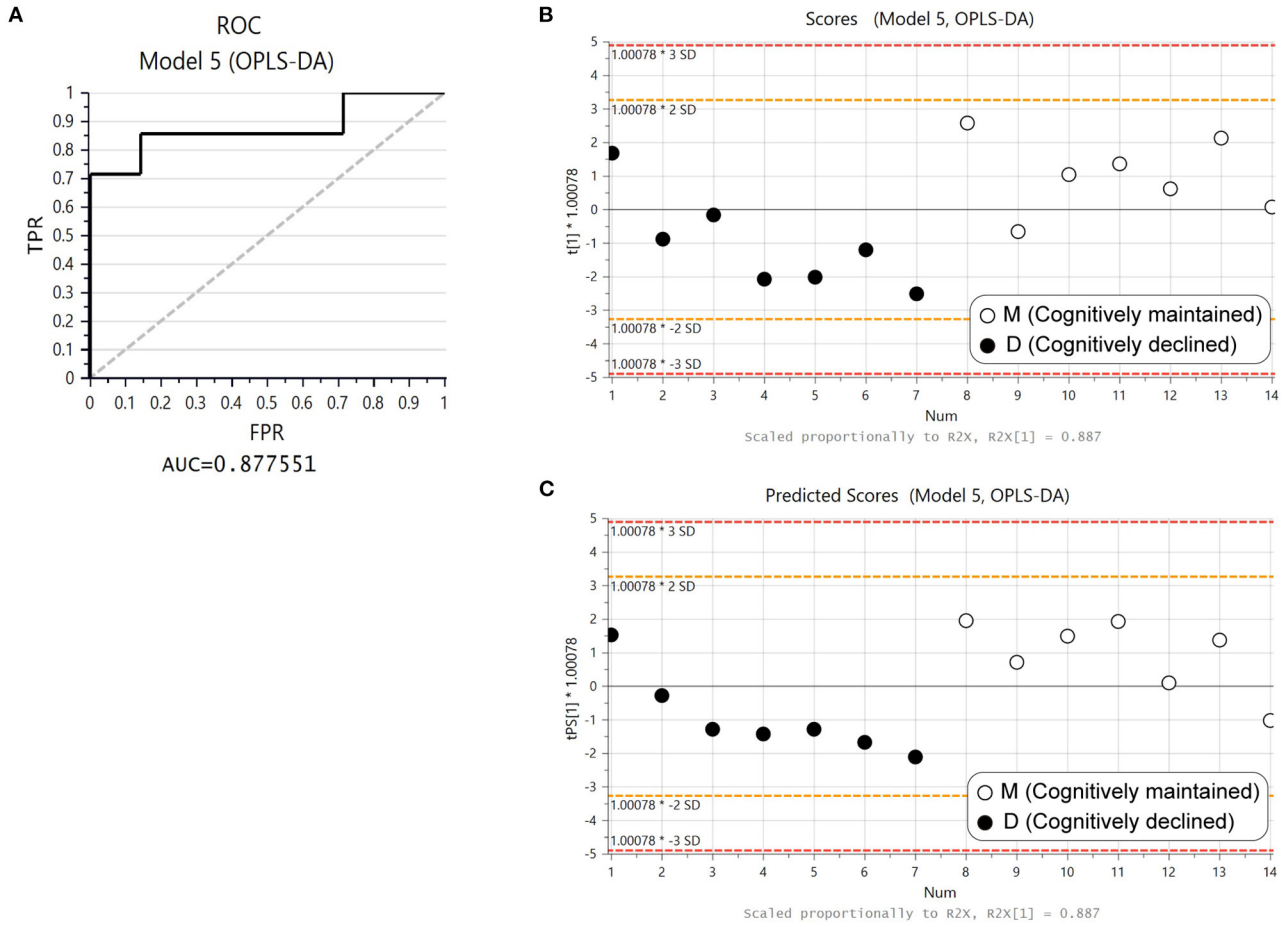
Predictive performance of ORM1, ORM2, and SERPINA3 with OPLS-DA. **(A)** Receiver operating curve (ROC) from the OPLS-DA model created with ORM1, ORM2, and SERPINA3 using baseline data (Model 5). **(B)** Score plot of Model 5. Solid circles: Group D1, open circles: Group M1. **(C)** Plot of predicted scores using follow-up data. Solid circles: Group D5, open circles: Group M5.

Three proteins (ORM1, ORM2, and SERPINA3) were included in both D1M1 and D5M5 discriminant models (Model 2 and Model 4). ORM1, ORM2, and SERPINA3 were identified in all urine samples, and their abundance was higher in Group D than in Group M at baseline and follow-up (Figure 2). An OPLS-DA model was created with these three proteins using baseline data as the test set (Model 5, 1 component, R^2^ = 0.42, Q^2^ = 0.33, *p* = 0.11 (CV-ANOVA)) and predictive analysis was performed with follow-up data as the prediction set. The predictive performance evaluated using ROC curve analysis showed an AUC of 0.878 (Figure 3A). OPLS-DA scores for the test set and prediction set of Model 5 were similar (Figures 3B, C). The proportions of correctly classified samples are shown in Supplementary Table 4, and the sensitivity and specificity of Model 5 for urine samples at both baseline and follow-up were 85.7 and 85.7%, respectively.

## 4. Discussion

We conducted cognitive function tests and collected urine samples from elderly community residents at two time points with an interval of ~5-years, and analyzed the urinary proteome prospectively and cross-sectionally between subjects with cognitive decline (≥4-point decline in MMSE score) and age- and sex-matched subjects who maintained normal cognitive function (MMSE score of 28–30 points). Both the prospective (i.e., comparison of urine proteomes at baseline) and cross-sectional (i.e., comparison of urine proteomes at follow-up) analyses generated models that significantly discriminated between Groups D and M. We identified 3 proteins (ORM1, ORM2, and SERPINA3) that were shared in both models. The discriminative model based on the 3 proteins, which was generated using proteomics data at baseline, had an AUC of 0.878 based on ROC curve analysis and showed similarly high sensitivity and specificity at both baseline and follow-up.

Although this is a pilot study with a small sample size, to our knowledge, it is the first prospective analysis of urinary protein biomarkers that predict cognitive decline. Our findings highlight the potential of urinary protein biomarkers that can predict cognitive decline.

ORM, also known as alpha-1 acid glycoprotein (AGP), is an acute-phase protein synthesized mainly in the liver and secreted in plasma (27). ORM is a highly glycosylated protein, with a molecular weight varying from 37 to 54 kDa depending on the degree of glycosylation (27). ORM genes are clustered on chromosome 9, and there are two paralogs in humans, ORM1 and ORM2 (27). ORM constitutes 1–3% of plasma and the concentration of ORM1 is five-fold higher than the level of ORM2 (27). Our urine proteomics data revealed that ORM1 and ORM2 were also present in relatively high concentrations in urine, with ORM1 being higher in abundance than ORM2.

The concentration of plasma ORM is known to increase 2–6-fold in most disease states, including inflammation and cancer (28). Therefore, ORM has been studied as a potential blood biomarker for many diseases (28–31). ORM has also been studied as a single or combined urinary biomarker of inflammatory diseases and cancer, including lupus nephritis, adult-onset Still's disease, psoriasis, rheumatoid arthritis, sepsis, Crohn's disease, bladder cancer, and hepatocellular carcinoma (32–38). In one cohort study, urinary ORM showed a significant association with incident hypertension at the 7-year follow-up (39). Hypertension, particularly in midlife, is associated with a high risk of dementia, including Alzheimer's disease, although these associations are not fully elucidated (40).

SERPINA3, or alpha-1-antichymotrypsin, is a member of the serine-protease inhibitor (SERPIN) superfamily. SERPINA3 is a highly glycosylated protein with a molecular wight of roughly 46kDa (41). SERPINA3 is an acute-phase protein that is mainly synthesized in the liver and secreted into the plasma. Altered expression of SERPINA3 in plasma, organs, cerebrospinal fluid, and urine is reportedly associated with various inflammatory diseases and cancers (41).

Dysregulation of SERPINA3 has been reported to be associated with Alzheimer's disease (42). Overexpression of SERPINA3 in the brain of AD patients correlates with tau hyperphosphorylation and senile plaque deposition (42). Recently, Vanni et al. reported that SERPINA3 expression in the brain is increased during aging and highly upregulated in patients with neurodegenerative disorders such as prion disease and Alzheimer's disease (43, 44). Cognitive decline is one of the most important signs preceding the onset of Alzheimer's disease. The increase in urinary SERPINA3 preceding cognitive decline observed in the present study is consistent with the findings of these previous studies.

All 3 proteins suggested as potential biomarkers of cognitive decline in our study were acute phase proteins and are known to be involved in various diseases such as cancer and inflammatory diseases as described above. Systemic chronic inflammation is one of the features associated with aging (45, 46). Hypertension is a common condition in the elderly population (47), and the prevalence of cancer and inflammation-related chronic diseases increases with aging (46). Given the limited sample size of this study, it should be considered that individuals with such conditions may not have been included in the control group. On the other hand, studies have identified inflammation as a potential driver of neurodegenerative brain changes and cognitive decline (48).

This study has some limitations worth noting. First, the urine proteome varies intra- and inter-individually based on factors such as age, sex, hormones, diet, and exercise (49). However, due to the study setting, it was not possible to restrict the timing of, or the conditions prior to, urine sampling. Therefore, inter-individual variation may have been high. Second, in this study, a decline of 4 or more points in MMSE score was considered a significant decline in cognitive function. However, the decline in MMSE score adopted in the study does not mean that the subjects have been diagnosed with some form of dementia. Lack of clinical diagnosis may result in a mixture of cognitive decline from different causes. Third, ORM1 is known to interact with various drugs (50), but no medication history was collected from subjects. Fourth, the possibility of selection bias must be considered. In selecting the control group, sex and age were matched to group D, but not MMSE score at baseline, and the control group was required to have a MMSE score of at least 28 at baseline. Mean MMSE scores at baseline tended to be statistically lower in group D. Finally, although the study was prospective in design and had a follow-up period of >4 years, the sample size was limited. However, to the best of our knowledge, this is the first exploratory study of urinary protein biomarkers of dementia using mass spectrometry in a longitudinal setting.

Prospective studies with larger sample sizes of cognitively normal elderly individuals, including those with urinary protein patterns similar to those shown in this study, would be warranted to explore urinary biomarkers that predict cognitive decline and/or dementia.

## Data availability statement

The original contributions presented in the study are publicly available. This data can be found here: Japan ProteOme STandard Repository (jPOSTrepo), https://repository.jpostdb.org/entry/JPST001969.

## Ethics statement

The studies involving human participants were reviewed and approved by the Human Research Ethics Committee of Niigata University. Written informed consent for participation was not required for this study in accordance with the national legislation and the institutional requirements.

## Author contributions

YW, YH, KKi, TY, and KN contributed to the study concept and design. YW, KKi, and KN contributed to the acquisition of data. YW, YH, and TY contributed to analysis and interpretation of data. YW and YH drafted the manuscript. YW, YH, KKi, TY, KKa, and KN critically edited the manuscript for important intellectual content. All authors contributed to the article and approved the submitted version.
