# Supplementary material for: Urinary proteome profiles associated with cognitive decline in community elderly residents—A pilot study
Source: Front Neurol. 2023 Mar 16;14:1134976. doi: 10.3389/fneur.2023.1134976 (PMC10061132; doi:10.3389/fneur.2023.1134976)
Supplement: Supplementary file 3 [file Image_1.pdf]

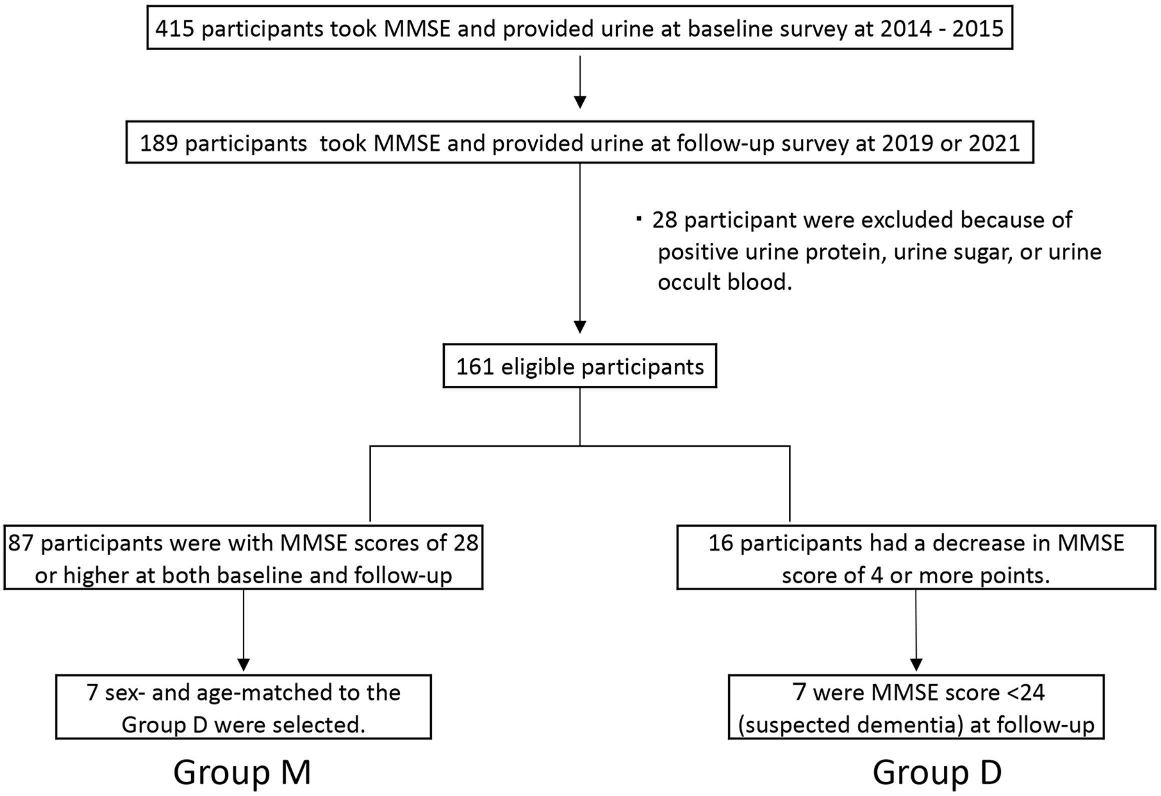

Supplementary Figure S1. Flowchart of participant selection

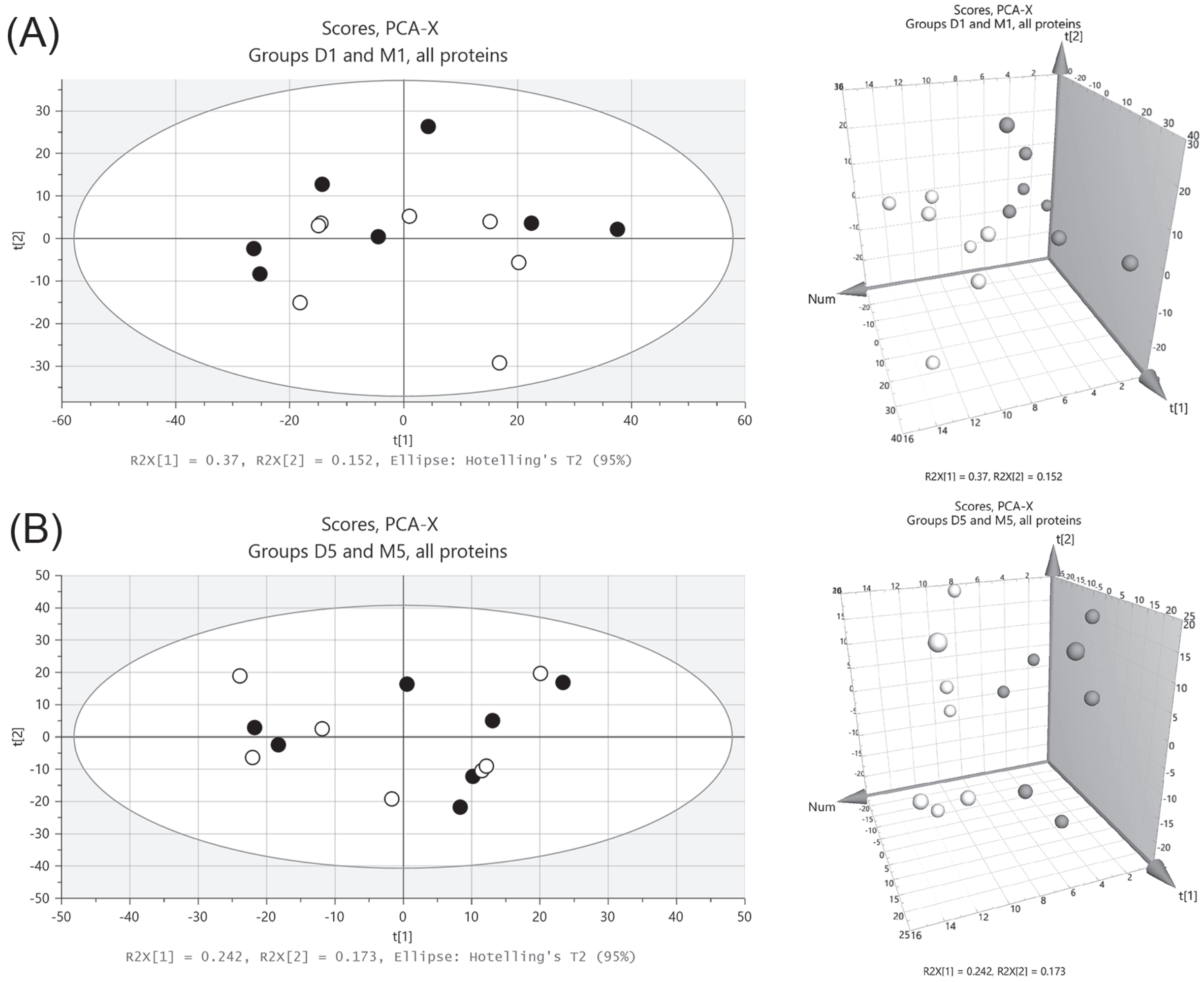

Supplementary Figure S2

Score plots of PCA. (A) PCA of baseline dataset. (B) PCA of follow-up dataset. The right panel of the figure shows the respective 3D charts. Black or grey spheres: Group D, open or white spheres: Group M.
